# Simultaneous Liver-Kidney Transplantation: Impact on Liver Transplant Patients and the Kidney Transplant Waiting List

**DOI:** 10.1007/s40472-018-0175-z

**Published:** 2018-01-19

**Authors:** Clifford D. Miles, Scott Westphal, AnnMarie Liapakis, Richard Formica

**Affiliations:** 1 Department of Internal Medicine, Division of Nephrology, University of Nebraska Medical Center, Omaha, NE USA; 2 Department of Internal Medicine, Section of Hepatology, Yale University School of Medicine, New Haven, CT USA; 3 Department of Internal Medicine, Section of Nephrology, Yale University School of Medicine, New Haven, CT USA

**Keywords:** Simultaneous liver-kidney transplantation, Organ allocation policy, Multi-organ transplant

## Abstract

**Purpose:**

The number of simultaneous liver-kidney transplants (SLKT) performed in the USA has been rising. The Organ Procurement and Transplantation Network implemented a new policy governing SLKT that specifies eligibility criteria for candidates to receive a kidney with a liver, and creates a kidney waitlist “safety net” for liver recipients with persistent renal failure after transplant. This review explores potential impacts for liver patients and the kidney waitlist.

**Recent Findings:**

Factors that have contributed to the rise in SLKT including Model for End-stage Liver Disease (MELD)-based allocation, regional sharing for high MELD candidates, and the rising incidence of non-alcoholic steatohepatitis will continue to increase the number of liver transplant candidates with concurrent renal insufficiency. The effect of center behavior based on the new policy is harder to predict, given wide historic variability in SLKT practice.

**Summary:**

Continued increase in combined liver/kidney failure is likely, and SLKT and kidney after liver transplant may both increase. Impact of the new policy should be carefully monitored, but influences beyond the policy need to be accounted for.

## Introduction

Renal failure after orthotopic liver transplantation (OLT) is an important risk factor for poor overall survival [1]. Moreover, pre-OLT renal dysfunction is predictive of post-OLT renal failure [2, 3]. Simultaneous liver-kidney transplantation (SLKT) has thus been employed as a treatment modality for individuals with end-stage liver disease (ESLD) and renal dysfunction, as a means to abrogate this risk. Since the introduction of the Model for End-Stage Liver Disease (MELD) score for purposes of deceased-donor liver allocation, which is heavily influenced by serum creatinine, there has been a substantial increase in the number of SLKT performed in the USA [4].

Concern has arisen surrounding the practice of SLKT, at least in part due to the absence of any specific policy addressing the allocation of the deceased-donor kidneys in the context of simultaneous renal-non-renal organ transplantation. Prior to August 2017, there were no existing formal SLKT criteria, such that centers could indicate the “need” for a kidney to be transplanted with a liver at their own discretion, including in situations where actual survival benefit from SLKT versus an isolated liver transplant was unclear [5], or when native renal recovery was likely. Organ procurement organizations (OPO) historically allocated the liver according to the liver match run, and if so indicated, the kidney would accompany the liver, presuming the candidate was listed in the same donor service area as the OPO. Note that this remains true for other combinations, such as heart-kidney and lung-kidney—the kidney “follows” the primary organ according to that organ’s match run. As has been reviewed previously, this practice is counter to the Organ Procurement and Transplantation Network (OPTN) Final Rule, primarily as it occurs in the absence of standardized medical criteria and is based largely on geographic proximity between donor and recipient [6•].

In response to the above concerns with SLKT, the OPTN organized a Working Group in 2014 to resume development of policy language to address the allocation of organs to individuals with ESLD and renal dysfunction. This Working Group was comprised of members of multiple OPTN committees, including Kidney Transplantation, Liver and Intestinal Transplantation, OPO, Ethics, Minority Affairs, and Operations and Safety, and built on a well-documented basis of work in this realm [4, 7, 8]. The policy language developed through data review, discussion, deliberation, and compromise was ultimately ratified by the OPTN Board of Directors in June 2016, and was implemented August 10, 2017. The details of the SLKT allocation development have been extensively reviewed elsewhere [6, 9, 10], and the policy itself can be found online on the OPTN’s website [11]. In brief, the new policy outlines medical eligibility criteria for adult candidates to receive a kidney transplant concurrent with a liver transplant, delineated by the presence of chronic kidney disease (CKD), acute kidney injury (AKI), or select metabolic diseases (Table 1). The SLKT policy also includes a “safety net” for *all* recipients of OLT who do not recover renal function after OLT, or subsequently develop advanced, persistent renal dysfunction within 60–365 days of transplant. Safety net candidates are assigned significant allocation priority in the kidney allocation system in order to receive an expedited kidney after liver transplant, appearing ahead of other local adult candidates [12]. Now that the meetings, debates, negotiations, and voting have all been completed, what can be the anticipated effect of this new policy on liver patients, and on the kidney transplant waiting list?

**Table 1:** Medical eligibility criteria for simultaneous liver-kidney transplantation. A transplant nephrologist must confirm that candidates meet *one* of the below criteria in order for them to receive SLKT organ offers

Chronic kidney disease (defined by having a glomerular filtration rate (GFR) ≤ 60 ml/min for at least 90 days), *and* at least one of the following: • The most recent GFR ≤ 30 ml/min • Has initiated chronic dialysis for end-stage renal disease (ESRD)
Acute kidney injury, as evidenced by having a combination of *either* of the following for 6 consecutive weeks (must be documented every 7 days): • Requiring acute dialysis treatment • GFR ≤ 25 ml/min
Metabolic disease: • Hyperoxaluria • Atypical hemolytic uremic syndrome (aHUS) due to mutation of either factor H or factor I • Familial non-neuropathic systemic amyloidosis • Methylmalonic aciduria

## Frequency of SLKT

**Fig. 1:**
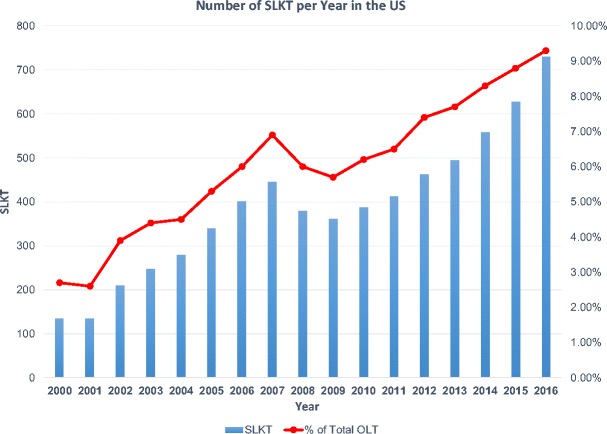
The number of SLKT performed in the USA has grown from fewer than 150 per year to more than 700 over the past 15 years. The percent of total liver transplants performed as SLKT has also risen four-fold over this time

One realm in which impact of the new SLKT policy is anticipated concerns the absolute number of SLKT that occur. As indicated in Fig. 1, the number of SLKT performed in the USA has been increasing, and exceeded 700 in the year 2016. In addition, the relative proportion of liver transplants performed as part of an SLKT has increased steadily, from 2.7% in 2000 to 9.3% in 2016. As serum creatinine is a prominent variable in the MELD equation, patients receiving liver offers have had an increasing burden of renal dysfunction since the adoption of MELD-based allocation policy. This was demonstrated early after MELD adoption, with an increase in proportion of liver transplant recipients having serum creatinine ≥ 2.0 mg/dl from 7.9% in April 1999 to 10% in December 2004 [5]. Additional policy change has prioritized sicker patients for broader regional sharing: “Share 35,” implemented in June 2013, dictated regional sharing of liver grafts to candidates with a MELD score of 35 or higher, as data analysis had indicated waitlist mortality in this subgroup of patients was comparable to that of status 1 patients [13]. The overall median MELD scores at transplant increased from 27 to 28, with scores in the post-implementation era ranging from 25 to 35 across OPTN regions [14]. In the pre-implementation era, 18.5% of OLTs were performed in candidates with MELD > 35, which increased to 26.5% post-implementation of “Share 35.” Since adoption of this expanded regional sharing for higher MELD candidates, there has been continued increase in the percentage of SLKT, from 7.7 to 9.3% of total OLT. Thus, MELD-based allocation and “Share 35,” with the continued increase in number of candidates on the liver waitlist, have contributed to a rising absolute number *and* proportion of SLKT.

The newly adopted SLKT allocation policy represents another influence on this process, though it is difficult to predict whether the net influence of the policy itself will be upward or downward on the number of SLKT performed. Indeed, as reviewed by Nadim et al., there has existed considerable regional variability in listing practices for SLKT. In their survey of US transplant centers, 25% used a CKD GFR cutoff of 40 ml/min as an indication for SLKT, and one third of programs used an AKI dialysis duration of 4 weeks [15•]. Going forward, some centers, particularly those with aggressive SLKT utilization, will clearly have fewer candidates eligible than they historically listed, while other programs who had been more restrictive may actually expand their listing. It has been estimated that 19% of SLKT recipients over a recent 10-year period would not have met the new eligibility criteria [10], but the literature lacks an estimate of how many more OLT recipients could potentially have been eligible, yet received an isolated liver transplant. Important to predicting the number of SLKTs that may occur going forward is the fact that the new policy sets minimum medical eligibility criteria, but does not require that candidates who meet them be listed for or receive an SLKT. It is conceivable that some centers will reflexively list and transplant all eligible candidates for SLKT, while others will continue to use more discretion, particularly with the availability of the safety net for those with renal non-recovery. Furthermore, the policy requires that a transplant nephrologist verify that candidates meet the eligibility criteria. Operationally mandating involvement of nephrology will likely have an impact on listing practices, and may tend to drive downward the proportion of candidates approved by centers for SLKT.

Changes in the ESLD population will also play a significant role in the frequency of SLKT. In 2015, Wong et al. reported that the number of adults with non-alcoholic steatohepatitis (NASH) awaiting liver transplant had almost tripled from that in 2004 and that NASH had become the second-leading disease among liver transplant waitlist registrants [16•]. More recently, Cholankeril et al. confirmed that NASH is the most rapidly growing indication for OLT in the USA [17]. Furthermore, the presence and severity of non-alcoholic fatty liver disease (NAFLD) were associated with an increased risk and severity of CKD in a recent meta-analysis, and thus there is an anticipated increase in the burden of CKD in liver transplant candidates [18]. In fact, Singal et al. reported that over a decade, NAFLD accounted for a significant increase in SLKT, rising from 8.2% in 2002 to 22% in 2011, while the proportion of transplants performed for hepatitis C (HCV) or alcohol-related liver disease dropped from 52 to 40% [19]. With advances being made in direct-acting anti-viral therapy for HCV, and the majority of treated patients now achieving sustained virologic response, the frequency of HCV-related ESLD and renal failure is expected to decrease. While HCV does cause renal dysfunction in some patients, the growth in number of liver transplant candidates with NASH and concurrent CKD will outpace any decline related to control of HCV.

On the whole, there will likely be a continued increase in the absolute number of SLKTs performed in the USA following enactment of the new OPTN SLKT allocation policy based upon the changing demographics of ESLD. However, caution must be exercised when drawing conclusions about causality based on the sheer number. The policy will likely reduce the proportion of liver transplant candidates listed for SLKT—in part due to the fact that some candidates who historically would have been listed for SLKT will simply not be eligible. The trend toward transplanting at higher MELD scores, though, is unlikely to change, and these patients by definition are more likely to have renal dysfunction. Also, the relative proportion and absolute number of liver transplant candidates with NASH as the etiology of cirrhosis are likely to continue their climb. The burden of CKD in this growing population will likely be the dominant force, and will drive the number of SLKT upward. Thus, in coming years, if the current trend is not attenuated, there may be as many as 800–1000 deceased-donor kidneys annually allocated to SLKT candidates, representing 6–7% of deceased-donor kidneys effectively removed from the pool available to the kidney-alone waitlist.

In addition to the sheer number of SLKTs occurring, the kidney community is affected by *which* deceased-donor organs are allocated as such. Historically, kidneys allocated for SLKT have generally been “higher-quality” organs than those transplanted as kidney alone. For example, Reese et al. showed that the mean Kidney Donor Profile Index (KDPI) for kidneys transplanted as SLKT was 36%, compared to 46% for kidney alone [20]. In other words, roughly half of the kidneys allocated “above” the kidney-alone match run to SLKT recipients are those that the Kidney Allocation System (KAS) is designed to preferentially allocate to pediatric kidney candidates—projected to number 400–500 per year. Similarly, Cheng et al. noted that 33% of the donor kidneys in their paired analysis had a KDPI < 20%, a subset of kidneys that KAS intends to allocate preferentially to kidney candidates with the longest expected post-transplant survival [21]. Thus, SLKT, as well as other renal-non-renal multi-organ transplants, will continue to concentrate donated kidneys with poorer expected performance in the remainder pool allocated to kidney-alone candidates. The organ transplant community will need to consider whether allocation priority schemes should take such issues into consideration.

## Safety Net Implications

The implementation of a safety net to allow accelerated access to kidney transplantation for OLT patients with persistent renal failure is an important component of the new SLKT policy. Liver transplant recipients who require chronic renal replacement therapy, or develop persistent CKD with a glomerular filtration rate (GFR) ≤ 20 ml/min, are eligible for safety net allocation priority when registered for a kidney transplant between 60 and 365 days after liver transplant. Safety net eligible patients will be prioritized above the local adult kidney waiting list population for donor kidneys with a KDPI > 20%, although safety net priority will remain below other prioritized groups including highly sensitized patients, 0-ABDR mismatches, prior living donors, and pediatric patients [12]. Additionally, safety net allocation priority does not apply to the highest quality deceased-donor kidneys (KDPI ≤ 20%). Justification for the adoption of a safety net includes its aim to improve the survival of early post-OLT recipients with renal failure via expedited kidney transplantation. Since OLT recipients with severe renal dysfunction or dialysis dependence have significantly impaired survival [22] and high waitlist mortality on the kidney transplant waiting list [23], physicians may have erred on the side of caution and listed patients for SLKT when there was uncertainty regarding native renal recovery prior to the existence of the safety net. However, past analyses have shown that patients who receive a timely kidney after liver transplant (KALT), within 1 year after liver transplant, achieve similar overall survival rates compared to SLKT recipients [8]. On the other hand, rejection-free kidney graft survival may be higher in SLKT recipients, suggesting a possible immunoprotective effect observed only when receiving a liver from the same donor as the kidney [24]. The safety net is intended to alleviate the burden of “guessing incorrectly,” and incentivize isolated OLT when there remains potential for renal recovery. It remains uncertain to what degree the safety net will contribute to transplant center behavior modification and SLKT listing practices for patients who may meet the minimum eligibility criteria, yet have reasonable potential for renal recovery.

Historically, a small minority of patients have been listed and transplanted within 1 year of OLT, as the majority of KALTs occurred several years later [25•]. According to OPTN data, from 2005 to 2013, 361 patients were registered for KALT within 1 year of OLT, an average of 40 per year [26]. During that same time period, only 93 patients actually received a KALT within the first post-OLT year, and of these, only 57 came from deceased donors (average 6.3 per year) with the remaining coming from living donors [26]. While the use of early deceased-donor kidney transplantation has previously been infrequent following OLT, with negligible impact on the total deceased-donor kidney pool, predicting the expected utilization of early KALT under the new safety net is challenging. The priority given via the safety net translates into shorter waiting times until kidney transplant for those candidates. Thus, it is likely that a higher percent of patients who are listed under the safety net will survive to kidney transplant. Further, while persistent renal failure following OLT is a relatively uncommon event overall [3, 27], there will likely be an increase in patients with post-OLT renal failure following the adoption of the medical eligibility criteria, as the number of ESLD patients with renal dysfunction that receive an isolated OLT increases. As with the total SLKT volumes, the number of candidates eligible for kidney priority via the safety will be reflective of continued increase in OLTs done for patients with NASH, obesity, diabetes, and other comorbidities that increase the risk of post-OLT renal failure [28•, 29]. As such, if the safety net leads to a substantial increase in early deceased-donor KALT, it is conceivable that any decrement in the number of SLKT due to the new eligibility criteria may be offset by rising numbers of KALT. It seems nearly certain that between SLKT and safety net prioritization for KALT, we will see a net overall increase in deceased-donor kidneys being allocated to liver transplant patients.

## Role of Living Donor Kidney Transplantation

It is important to consider the role that living kidney donation could play in this arena. In many ways, living kidney donation has always provided a “safety net” to liver transplant recipients with renal failure and an available donor. Knowledge of a willing and acceptable living kidney donor can and should inform the decision of whether a liver transplant candidate with renal dysfunction should be considered for SLKT. From an organ-shortage vantage point, centers should be reluctant to list candidates for SLKT in the presence of a viable living donor. For safety net situations, candidates should be encouraged to pursue living donation despite the relative list priority afforded them by the new policy. There will likely exist some impetus to await a deceased donor because the expected waiting time will be “short,” but in reality, it is difficult to predict how long a given candidate will wait once they have met safety net priority listing criteria. The waiting time will undoubtedly be affected by geography, sensitization, and blood type, among other things. Such candidates should be counseled about the relative survival benefit of receiving a living donor kidney transplant compared to a deceased donor [30]. A discussion of the relative benefits should include the fact that the safety net provision does *not* include priority for kidneys with KDPI ≤ 20% (“Sequence A”), and that candidates are unlikely to receive a deceased-donor kidney that exceeds the quality of a living donor kidney. Further, living donor kidney transplantation could be done at any time deemed appropriate by the transplant team, not being bound to the time constraints of the policy.

## Conclusion

In addition to the likelihood of having more kidneys removed from the pool that is allocated to “kidney-alone” recipients, those transplanted as either SLKT or via the safety net will be disproportionately higher-quality organs (lower KDPI). Thus, some degree of decline in outcomes for this non-liver-kidney transplant population should be anticipated. This policy also does not alter the fact that multi-organ transplant candidates, including SLKT, continue to receive priority above *all* kidney-alone transplant candidates, even those groups prioritized by KAS, such as pediatric patients, prior living donors, and those that are highly sensitized. The performance of this policy will be scrutinized by many, and that scrutiny will clearly include numeric counts of donor kidneys that are transplanted as SLKT and as safety net transplants to prior OLT recipients, and the incidence of living donor KALT. Additionally, it is important to ensure that the relative donor quality is reported across the spectrum of SLKT, safety net, and kidney-alone transplants. Waitlist, graft, and patient outcomes should be compared for the affected groups (SLKT, isolated OLT, safety net, and kidney-alone recipients). This policy and practice lie at a complicated intersection of a medical urgency-based allocation system (liver) and a largely utility-based system (kidney). Inferences, conclusions, and further policy revisions will necessarily be difficult to come by, but all should be informed by meticulous collection and analysis of data. Beyond SLKT, refinements need to be made to other multi-organ allocation situations, such as heart-kidney, lung-kidney, and heart-liver. The transplant community is watching carefully as this important step is taken into codification of multi-organ transplantation.
